# Resveratrol’s neural protective effects for the injured embryoid body and cerebral organoid

**DOI:** 10.1186/s40360-022-00593-3

**Published:** 2022-07-12

**Authors:** Yanli Wang, Tingting Wei, Qiang Wang, Chaonan Zhang, Keyan Li, Jinbo Deng

**Affiliations:** NHC Key Laboratory of Birth Defects Prevention, Henan Key Laboratory of Population Defects Prevention, Henan Institute of Reproduction Health Science and Technology, Zhengzhou, 450002 Henan Province China

**Keywords:** Neuroprotection, D-galactose, Resveratrol, Embryoid body, Cerebral organoid, HiPSCs, 3D culture

## Abstract

**Objective:**

Resveratrol (RSV) is a polyphenol compound found in grapes, veratrum and other plants. It has been reported that RSV has anti-inflammatory, anti-oxidant, anti-cancer and other pharmacological effects. However, the impacts of RSV on development of nervous system are not understood well. The study aims to investigate RSV’s neuroprotective effect during development and to provide a health care for pregnant women and their fetuses with RSV supplementation.

**Methods:**

In this study, we induced human induced pluripotent stem cells (hiPSCs) to form the embryoid bodies (EBs) and cerebral organoids (COs) with 3 dimensional (3D) culture. In the meantime, D-galactose (D-gal, 5 mg/ml) was used to make nervous injury model, and on the other hand, RSV with various doses, such as 2 μm/L, 10 μm/L, 50 μm/L, were applied to understand its neuroprotection. Therefore, the cultures were divided into control group, D-gal nervous injury group and RSV intervention groups. After that, the diameters of EBs and COs were measured regularly under a reverted microscope. In the meantime, the neural proliferation, cell apoptosis and the differentiation of germ layers were detected via immunofluorescence.

**Results:**

(1) D-gal could delay the development of EBs and COs; (2) RSV could rescue the atrophy of EBs and COs caused by D-gal; (3) RSV showed its neuroprotection, through promoting the neural cell proliferation, inhibiting apoptosis and accelerating the differentiation of germ layers.

**Conclusion:**

RSV has a neuroprotective effect on the development of the nervous system, suggesting RSV supplementation may be necessary during the health care of pregnancy and childhood.

## Introduction

Resveratrol (RSV, 3,4′,5-trihydroxystilbene), one of polyphenolic compound, presents in plants, including grapes, red wine, nuts, berries, reed rhizome and others. RSV consists of two aromatic rings and exists in cis- and trans- isomers. Only trans-isomer is useful and stable, exerting its biological activity [1], such as anti-inflammatory, anti-oxidation, anti-free radicals, anti-depressive [2], anti-bacterial, anti-cancer, anti-cardiovascular diseases, neuroprotective effects [3] and other biological activity [4, 5]. RSV has also been reported as a safe and non-toxic nutritional supplement and has been listed as a healthy food supplement by International Conference on Healthy Food. Many studies have reported that RSV is beneficial to embryonic development [6, 7]. However, whether RSV has a protective effect on the neural development has not yet been understood well, especially for human being. Fortunately, the 3D cultured embryoid bodies (EBs) and cerebral organoids (COs) derived from human induced pluripotent stem cells (hiPSCs) provide us a useful tool for human alternative experiment. The hiPSCs in the study are reprogrammed from urine exfoliated cells induced by transplanting stem cell transcription factors [8]. After 3D culture, hiPSCs have a tendency to spontaneously develop into certain primitive structures [9], such as EB.

In the study, with hiPSCs, the EBs and COs were 3D-cultured, then, the control group, the D-gal group and RSV group were divided randomly. Finally, we examined the EB’s development, the COs neural proliferation of neural stem cells, cell appotosis and the differentiation of three germ layer in various groups. Therefore, RSV’s neural protection on early developing nervous system could be investigated, and hopefully, the study will provide us some knowledge of RSV supplement during the health care of pregnancy and childhood.

## Results

### Normal development of EB and CO

**Fig. 1:**
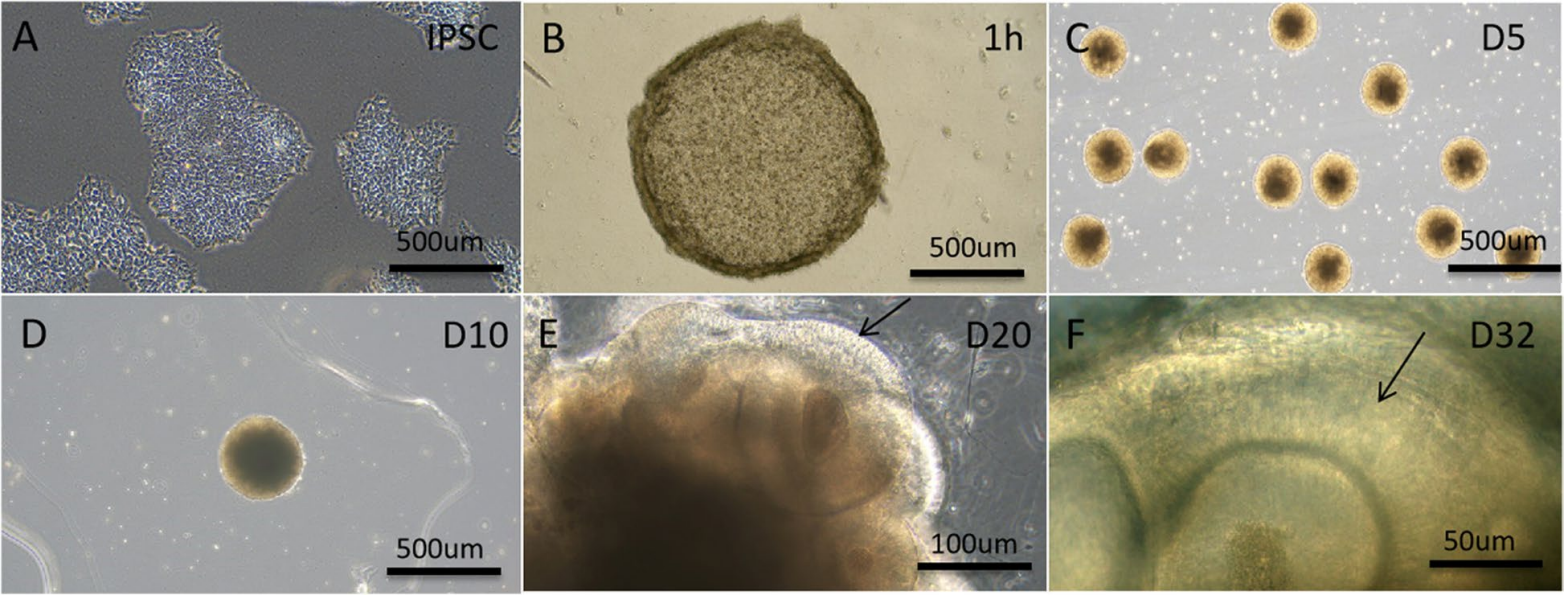
The development of cerebral organoid (COs). **A**, hiPSCs start to aggregate each other. **B**, hiPSCs gather together to form embryoid bodies (EB) at 1 hr. after plating. **C**, EBs cultured at day 5 (D5). **D**, at day 10 (D10), COs were transferred to Matrigel droplet. **E**, at day 20 (D20), preliminary CO can be found, and in the periphery of cultivation, some transparent annular buds, presumable NRs, appear (↑). **F**, at day 32 (D32), the cerebral lamination is formed in COs, the organoid volume increases and the radial epithelium becomes thick, showing the typically laminar structure (↑). **A**-**D**, scale bar = 500 μm; **E**, scale bar = 100 μm; **F**, scale bar = 50 μm

In order to understand well, it is necessary to describe the normal development of EB and CO. Initially, the hiPSCs trended to aggregate spontaneously to form cell masses called “clones” with clear boundaries (Fig. 1A). After transfer into 96-well U-bottom plate, the hiPSCs assembled further to form EBs (Fig. 1B). At day 5 (D5), the loose cells gathered tightly to form cell mass with uniform size, it was called EB (Fig. 1C). Subsequently, EBs after neuroepithelial induction grew and developed in Martrigel (Fig. 1D). After further culture for a few days, COs appeared like a ring with bright periphery and dark center. The buds with a cavity inside were named neural rosettes (NRs), they were important structure for the histogenesis of COs. At day 20 (D20), the buds became larger and larger, and the neural rosettes in periphery developed into preliminary COs (Fig. 1E). At day 32 (D32), the COs with radial structure became thick, showing the typical lamination as cortex (Fig. 1F).

### RSV intervention and EB development

**Fig. 2:**
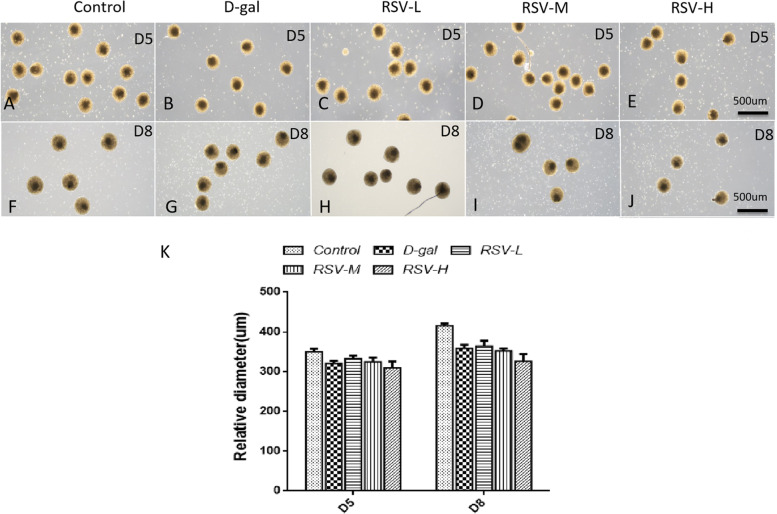
The changes of embryoid bodies (EBs) and cerebral organoid (COs) after resveratrol (RSV) treatment. **A**-**E** show the control group, D-gal group, RSV-L, RSV-M and RSV-H groups at D5 respectively. **F**-**J** show the control group, D-gal group, RSV-L, RSV-M and RSV-H groups at D8 respectively as well. Compared with control group (**A**, **F**) at D5 and D8, the size of spheres in the D-gal group (**B**, **G**) is decreased sharply. RSV can rescue the development of EB and COs delay, in RSV-L (**C**, **H**) groups at D5 and at D8 as well, and make the EB and COs’ size increased compared with that in the D-gal group (**B**, **G**), however, RSV’ rescue is not obvious in RSV-M (**D**, **I**) and RSV-H (**E**, **J**) group, comparing with RSV-L. **K**, statistical analyses for the EBs diameter are made. In the same age, RSV treatment can increased the size of spheres. **A**-**J**, scale bars = 50 μm; Control: the control group; RSV-L: the RSV low-dose group; RSV-M: the RSV medium-dose group; RSV-H: the RSV high-dose group

To observe the influence of RSV on EBs formation, EBs were 3D-cultured in EB formation media, with or without RSV intervention. In intervention groups, three different doses of RSV were treated, they were 2 μm/L, 10 μm/L, 50 μm/L respectively. The diameters of EBs and COs was measured using ImageView at D5, day 8 (D8) and D12. They were (350.5 ± 2.49) μm and (415.6 ± 2.70) μm at D5 and D8 respectively (P<0.05) in the control group (Fig. 2A & F). However, at D5, the EBs’ size was decreased at the D-gal group compared with the control group, due to its effect of EBs' injury. RSV seemed to rescue D-gal’s injury, for instance, in the RSV low-dose group (RSV-L), the EBs were bigger than in D-gal group (Fig. 2A-E), and the same phenomenon was observed in same groups at D8 as well (Fig. 2F-K).

### RSV intervention and neural proliferation

**Fig. 3:**
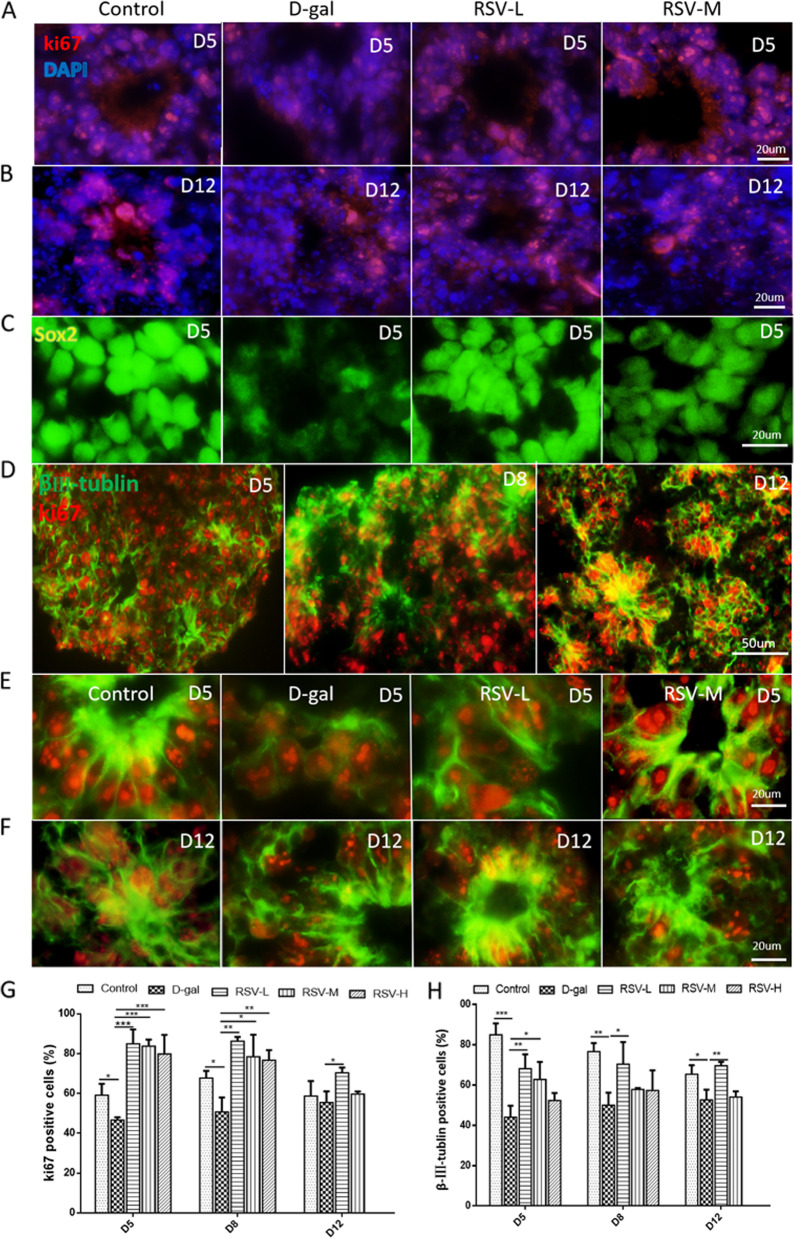
The neural proliferation in embryoid bodies and cerebral organoids after resveratrol (RSV) treatment. **A**-**B**, the ki67 (red) positive cells in each group, at D5, the cell number is decreased in the D-gal group compared to that in the control group, and RSV can rescue the cell’s decline, and the cells’ amount at D12 has the same trend as that at D5 (*p* < 0.05), DAPI (blue) was used as counterstaining. **C**, the expression of Sox2 (green) is similar to results as ki67 positive cells in various groups. **D**-**F**, β-III-tublin (green) and ki67 (red) immunolabeling. RSV can rescue the cells’ decrease. **E**-**F**, at D5 and D12, β-III-tublin-positive cells decrease in the D-gal group compared to that in the control group (*p* < 0.05), the amounts of β-III-tublin in the RSV-L increases significantly (*p* < 0.05), compared with that in D-gal group. **G**, **H**, statistical analyses show the ki67 and β-III-tublin-positive cells increased significantly in the RSV-L and RSV-M at D5, D8 and D12. **A**-**C** & **E**-**F**, scale bars = 20 μm; **D**, scale bars = 50 μm

In order to explore the effects of RSV on the neural proliferation in the EBs and COs, the proliferated cells were tested by ki67 and Sox2 immunolabeling. At D5, the percentage of ki67-positive cells (the numbers of ki67-positive cells/the numbers of total cells) were (59.13 ± 3.31)%, (46.64 ± 0.81)%, (84.93 ± 4.2)%, (83.78 ± 1.89) %, (79.82 ± 5.548)% in the control, D-gal, RSV-L, the RSV medium-dose group (RSV-M), the RSV high-dose group (RSV-H), respectively. The ki67-positive cells were decreased in the D-gal group compared with that in the control group, whereas after RSV rescue, the amount of ki67-positive cells were increased in the RSV groups compared with D-gal group, (Fig. 3A-B & G), and the same phenomenon was observed at D8 and D12. Meanwhile, the expression trend of Sox2-positive cells was similar with that of ki67 in each group (Fig. 3C).

As we know, neuroepithelium is an active place for neural proliferation. In order to observe RSV’s rescue the nervous injury during the development, β-III-tublin was used to label the microtubules in neuroepithelial cells of NRs. In this study, the β-III-tublin-positive neuroepithelial cells could be double-labeled with ki67, suggesting in the early age, these neuroepithelial cells were active for neural proliferation and neural differentiation. For instance, in control group, the β-III-tublin-positive cells were mainly distributed radially around the lumen of NRs. As the prolongation of culture time, the cavity of the NR gradually became smaller (Fig. 3D). the percentage of β-III-tublin-positive cells were (84.91 ± 3.29)%, (44.03 ± 2.88)%, (68.12 ± 3.59)%, (62.78 ± 4.351) %, (52.32 ± 2.17)% in the control, the D-gal, RSV-L, RSV-M, RSV-H at D5, respectively. The β-III-tublin-positive cells in the D-gal was significantly decreased compared with that in the control group (*p* < 0.05), and RSV could rescue the cell decline in RSV-L and RSV-M, compared with that in the D-gal group (*p* < 0.05), but the RSV-H (50 μm/L) was not very as effective as in RSV-L and RSV-M, and the same trend was observed at D8 and D12. (Fig. 3E-F & H).

### RSV intervention and cell apoptosis

**Fig. 4:**
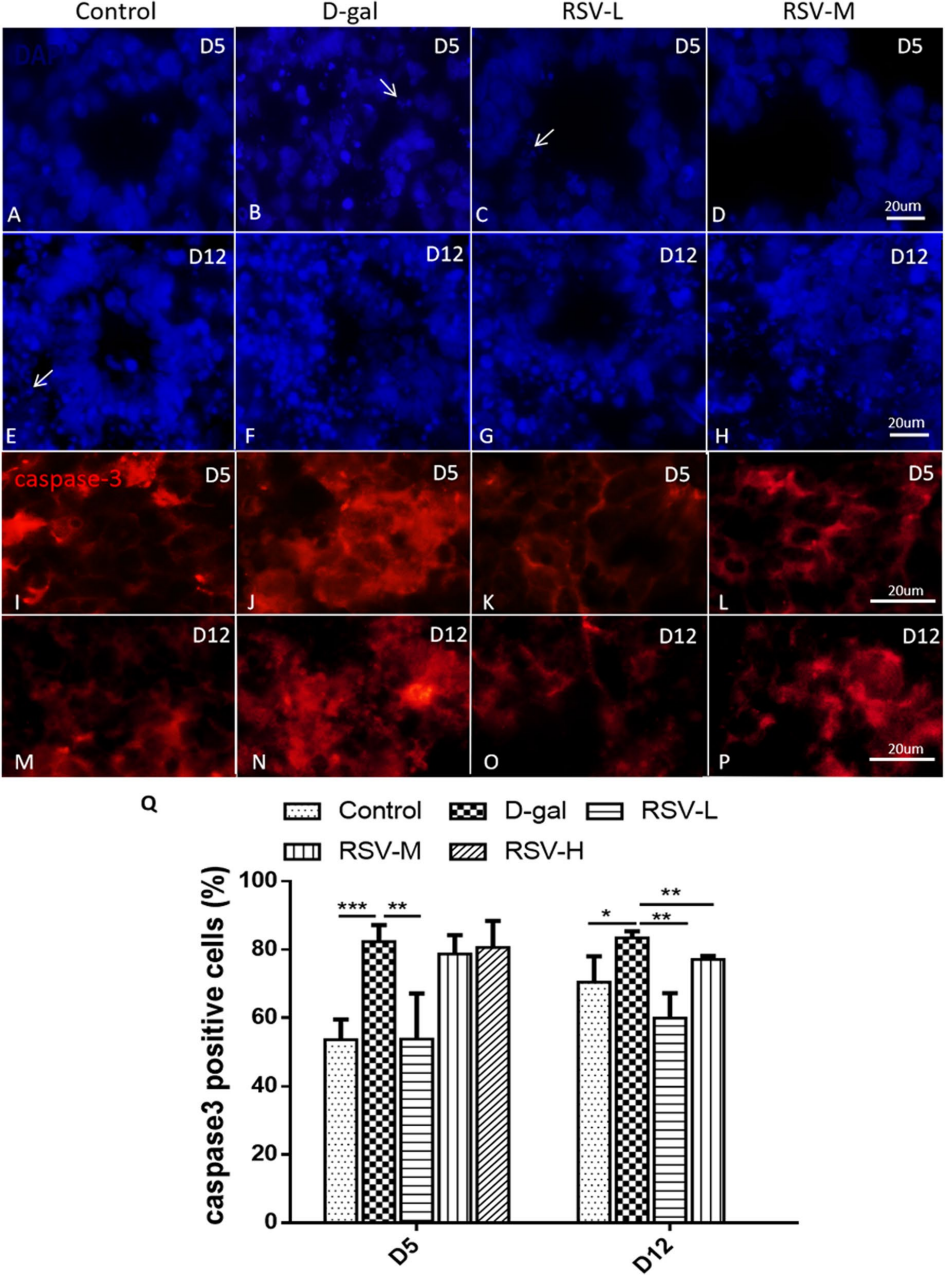
Cellular apoptosis after resveratrol (RSV) treatment. **A**-**H**, DAPI staining (blue) shows that the nuclear fragments (arrows) is increased in the D-gal group compared with that in the control group, meanwhile, the cell apoptosis is decreased in the RSV groups at D5 and D12. **I**-**P**, the caspase-3-positive cells (red) in various groups. Numerous apoptotic cells can be found in embryoid bodys and cerebral organoids of every group at D5 and D12 as well, but the caspase-3-positive cells in the D-gal group are maximum, compared with other groups, RSV can rescue cell apoptosis. **Q**, statistical analyses show caspase-3-positive cells are increased in the D-gal group compared with that in the other group, and RSV can rescue cell apoptosis significantly in the RSV groups. **A**-**P**, scale bars = 20 μm

Apoptosis is a kind of programmed cell death controlled by genes. DAPI staining shows that apoptotic cells appear apoptotic bodies with nuclear fragments. The caspase-3 can also be used to mark apoptotic cells. In this study, the effects of RSV on cell apoptosis was explored as well. Our results showed that D-gal could induce cell apoptosis, for instance, the apoptotic bodies in the EBs gradually increased in D-gal group, compared with that in the control. However, after RSV intervention, cell apoptosis decreased, compared with control and D-gal groups at D5 and D12 (Fig. 4A-H). The results of caspase-3 immunocytochemistry were similar to DAPI staining. The percentage of caspase-3-positive cells were (53.63 ± 3.42)%, (82.30 ± 2.43)%, (53.81 ± 6.69)%, (78.69 ± 3.22)%, (80.66 ± 3.88)% in the control, the D-gal, RSV-L, RSV-M, RSV-H at D5, respectively. D-gal could induced cell apoptosis, and RSV could rescue cell apoptosis of the EBs and COs in RSV groups, at also D12 (Fig. 4I-Q).

### RSV intervention and germ layer differentiation

With 3D structure, EB spontaneously differentiates into three germ layers according to the interaction between different cells [10], and the various cells in three germ layers can be labeled with various proteins. The ectoderm can develop into the body’s epidermis, nerves, lens and other structures. Nestin is an important marker for neural progenitor cells and early radial glial cells. Therefore, Nestin was used to label the ectodermal cells.

**Fig. 5:**
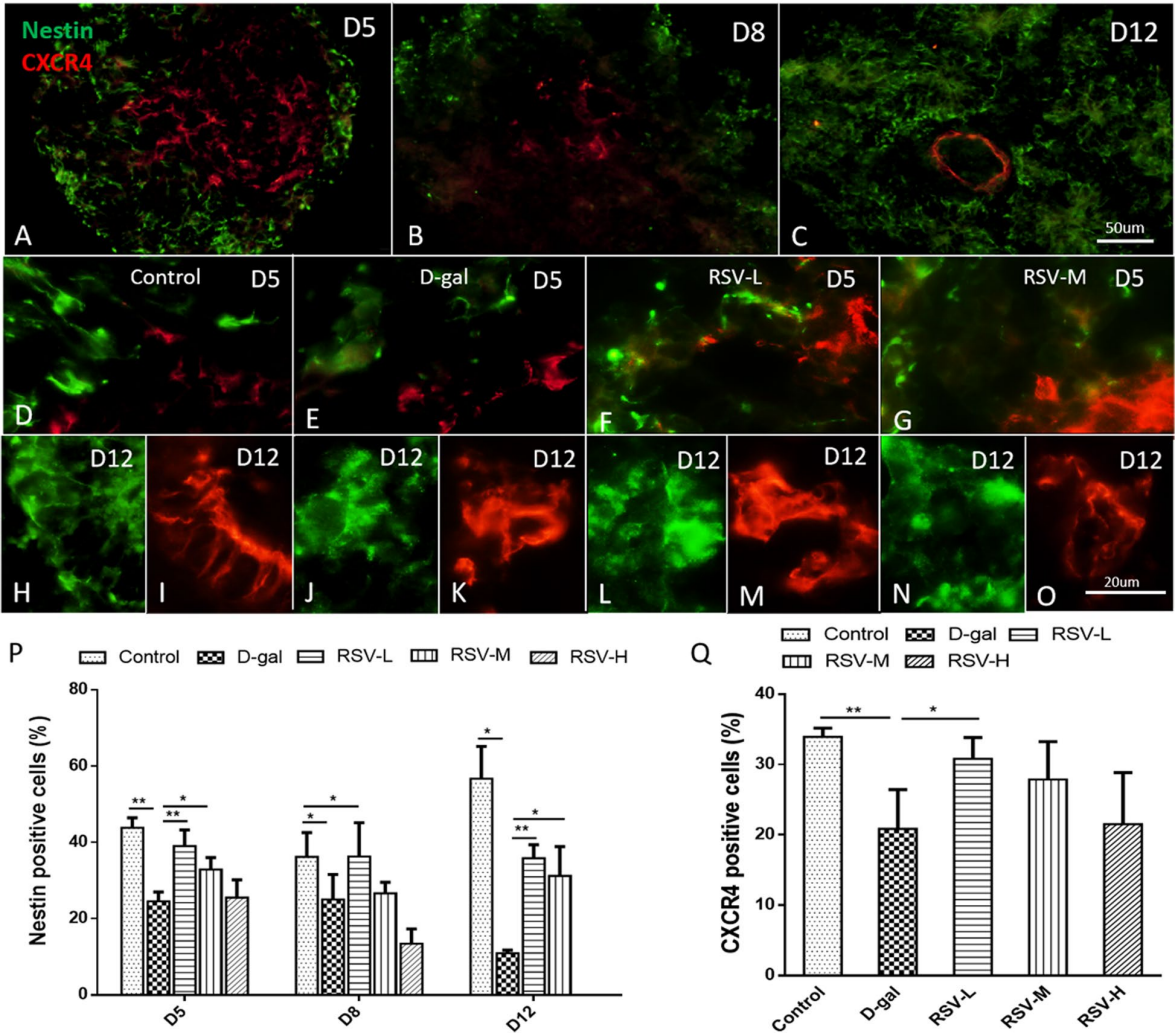
Germ layer differentiation after resveratrol (RSV) intervention. **A**-**C**, the amounts of Nestin (green) and CXCR4 (red) in vitro at D5, D8 and D12, Nestin-positive cells appear irregular filaments at D5. As the culture time increased, Nestin-positive cells regularly surround the NRs, and CXCR4-positive cells surround into regular circles in the embryoid bodys and cerebral organoids. **D**-**O**, the amounts of Nestin and CXCR4 in different groups in vitro at D5 and D12, Nestin and CXCR4 are expressed weakly in the D-gal compared with that in the control and RSV groups (*P* < 0.05), and RSV can restore the their expression in RSV-L, compared with D-gal group (*P* < 0.05). **P**, **Q**, statistical analyses show, at same age, the expression of Nestin and CXCR4 is weaker and less in the D-gal group compare with that in the control, and RSV can rescue the expression of Nestin and CXCR4 positive cell numbers (*P* < 0.05). **A**-**C**, scale bars = 50 μm; **D**-**O**, scale bars = 20 μm

The results showed that Nestin-positive cells existed largely in various stages of the EBs and COs, and they mainly located in the neural rosettes, the original locus of various types of neurons and glia (Fig. 5A-C). the percentage of Nestin-positive cells were (43.83 ± 1.51)%, (23.86 ± 1.80)%, (39.04 ± 2.42)%, (32.87 ± 1.571)%, (25.51 ± 2.351)% in the control, the D-gal, RSV-L, RSV-M, RSV-H at D5, respectively. so the ratio was significantly decreased in the D-gal compared with the control (*P* < 0.05), but after RSV intervention, the Nestin-positive cells could get restored in the RSV-L and RSV-M (*P* < 0.05) compared with that in the D-gal at D5 and at D12 as well. (Fig. 5D-O & P).

The mesoderm can develop into some tissues, such as blood vessels, bones and muscles. CXCR4 is involved in the formation of bone marrow and the transport of blood stem cells, so CXCR4 is often used to label mesoblastema. Our results showed that at D5, the percentage of CXCR4-positive cells were (33.92 ± 0.72)%, (20.84 ± 3.95)%, (30.80 ± 1.74)%, (27.88 ± 3.09)%, (21.50 ± 3.29)%, (21.5 ± 3.29)% in the control, the D-gal, RSV-L, RSV-M, RSV-H at D5, respectively. The expression of CXCR4 decreased in the D-gal compared with that in the control, but after RSV supplement, the expression of CXCR4 increased compared with that in the D-gal (Fig. 5D-O & Q). The results suggested that RSV played a positive role for the differentiation of germ layers.

### RSV intervention and the development of COs

**Fig. 6:**
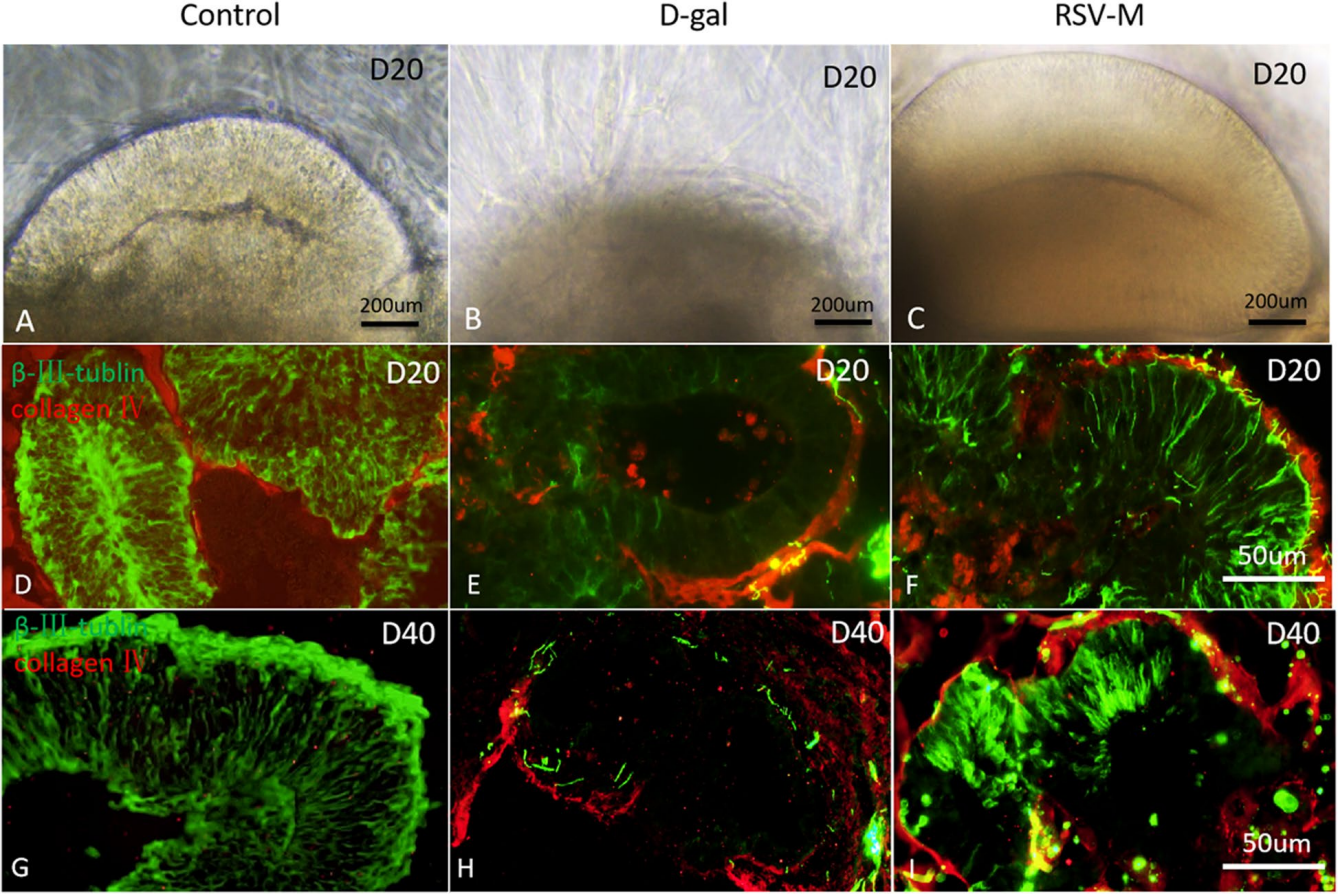
Differentiation of neuronal cells in cerebral organoids after resveratrol (RSV) treatment. **A**-**C**, at D20 in vitro culture, the cortical morphology of different groups under an inverted microscope, the NRs are thicker in the control group and the RSV-M, while the NRs in the D-gal are not observed. **D**-**I**, at D20 and D40, a large number of β-III-tublin-positive cells are distributed radially around the basal side of the lumen. A layer of collagen IV-positive basement membrane is wrapped around the NRs’ periphery, β-III-tublin-positive cells are decreased in the D-gal group compared with that in the control group, and β-III-tublin-positive cells are increased after RSV treated compared with that in the D-gal. **A**-**C**, scale bars = 200 μm; **D**-**I**, scale bars = 50 μm

We also studied the RSV’s effects on the development of **COs**. As the culture time prolonged, the NR’s walls gradually became thicker and developed into the cortex-like structure. At D20, NRs couldn’t even be found in D-gal group, but NRs appeared clearly in the control and RSV-M groups, under an inverted microscope (Fig. 6A-C). We used β-III-tublin and collagen IV to label neuroepithelium cells and the basal membrane of neuroepithelium in NRs. At D20 and day 40 (D40), a large number of β-III-tublin-positive cells were distributed radially around lumen in the control group, We observed that the β-III-tublin-positive cells decreased in the D-gal compared with control group, however, RSV could increase the β-III-tublin-positive cells’ number (Fig. 6D-I).

## Discussion

There is burgeoning interest in the use of natural compounds for possible therapy and health care. As a natural polyphenol compound, RSV has been shown to have many beneficial health properties, such as decreasing blood sugar [11], fighting cancer [12], antibacteria [13], cardioprotection etc. [14]. Recently, RSV can also suppress the nociceptive neuronal activity [15], and RSV also is used to treat dementia [16]. RSV exhibits beneficial effects in several neurological disorders such as Alzheimer disease [17], Parkinson disease [18], and amyotrophic lateral sclerosis [19]. RSV also has potential therapeutic effects on developmental neurotoxicity caused by long-term fluoride exposure [20]. Animal experiments have also suggested that RSV provides protective effects for embryos against teratogenic insults, such as exposures to ethanol, dioxin, and maternal diabetes [21]. In this study, the EBs were exposed to RSV at the concentrations of 0, 2, 5, 10, 20, 50 and 100 μm/L, the concentrations of RSV were screened according to the volume of cultures and the degree of cell death visually. Adverse morphogenetic effects were observed when EBs and COs were treated with RSV at 50um/L and higher, raising the possibility that in utero exposure to such concentrations would impair embryo development. The results of Kim et al. implicates RSV had potential developmental toxicity in EBs [22].

Fetal neural malformation is one of the most common birth defects in humans, and it is one of the main causes for the increase in perinatal morbidity and mortality. At present, folic acid is only used clinically to prevent neural tube malformations, there is no clinically effective medications to treat neural malformations. Therefore, the current prevention and treatment methods are far from reaching the actual clinical demands, so it is particularly important to research on the clinical application of medications that protect the development of the nervous system during the embryonic period. In order to understand RSV’s potential neuroprotective effects, we used the nerve injury model of EBs and COs to explore RSV’s rescue function during the early development of nervous system. In this study, we try to understand RSV’s neuroprotective mechanism further. According to our study, the neuroprotective mechanisms of RSV are as follows.

### RSV promotes the cells’ proliferation in EBs and COs

The structure and function of EBs cultured in 3D from hiPSCs are similar to blastocysts in the early stage of embryonic development [23]. In this study, we intuitively observed the effects of RSV on the growth of the EBs by measuring the diameter of the EBs and COs. The results showed that RSV can promote the increase of sphere sizes. For instance, we found that RSV promoted the neural proliferation and promoted the differentiation of ectoderm and mesoderm. Previous studies have found that some chemicals such as bisphenol A up-regulated the pluripotency marker Sox2 in mouse EBs at the mRNA and protein levels and increased cell proliferation [24]. The decrease of Sox2 expression up-regulated the differentiation regulator Cdx2 that leads to cell differentiation [25], which suggests that RSV may interfere with the differentiation of EBs by affecting the expression of pluripotent factors. Nestin was originally described in the neuroepithelial stem cells (NSCs) in the developing and adult brain, and now it is known that Nestin was expressed in tissues and stem cells in various forms, such as pancreatic islets [26], skeletal muscle satellite cells [27], testes [28], and hair follicles [29], Heart [30] and part of the bone marrow of non-hematopoietic cells [31].

During the development of the nervous system, the lack of Nestin protein can cause a large number of apoptosis of neural tube cells and hinder the formation of neural tubes in the ectoderm. The results of Gould [32] and other studies verified this phenomenon. In this study, Nestin-positive cells increased significantly after RSV treatment, which was conducive to the development of NRs and promoted the differentiation of ectoderm. Nestin-positive cells and their protrusions can also be seen around the NRs. During the initiation of early neurogenesis, Nestin-positive cells also represent radial glia [33], and radial glia cells are the precursor of neurons, astrocytes, and oligodendrocytes [34].

### RSV inhibits cell apoptosis in EBs and COs

Apoptosis plays a key role in embryonic development. It is a normal cell metabolism program that can effectively remove damaged cells. A large number of studies have shown that RSV can reduce cell apoptosis by regulating the expression of related genes. And there were studies showed that RSV significantly reduced the expression of caspase-3 protein and inhibited the apoptosis of oocytes [35–37]. This study found that caspase-3 protein expression decreased in the RSV can rescue the cell apoptosis induced by D-gal, indicating that RSV can inhibit cell apoptosis.

The most commonly cited RSV’s anti-apoptosis mechanism is that RSV is an activator of class III histone deacetylase sirtuin1 (SIRT1), which is a crucial regulator in the pathophysiology of neurodegenerative diseases [18, 38]. RSV can activate SIRT1 and then inhibit the expression of caspase-3, which is its possible anti-apoptosis mechanism. However, the specific anti-apoptosis mechanism needs to be further studied.

### RSV is possibly vital for the development of fetus and children

Studies have pointed out that [39] RSV can promote the maturation of bovine oocytes and subsequent embryonic development of in vitro fertilization and also improve sheep embryo development [40]. RSV supplementation may have a protective effect on fetal and childish neurodevelopment. Supplementation of RSV during pregnancy or early childhood should be considered to be helpful for brain development and cognitive behavior. In this way, we also suggest that RSV and RSV combined with other chemicals may have a positive impact on the cognitive barriers from neurodevelopmental disorders.

In conclusion, (1). RSV has neuroprotection effects on the development of the nervous system, through promoting neural development and differentiation, and inhibiting the cell apoptosis. (2). RSV supplementation probably becomes a potential nutrients for pregnant women and children.

## Materials and methods

### Cell culture

HiPSCs (UE017C1) were purchased from Guangzhou Institutes of Biomedicine and Health, Chinese Academy of Sciences were thawed and cultured with mTesR1 medium (Stem Cell, 85,850) in humidifed 37 °C and 5% CO2 incubators on hESC qualified Matrigel (BD, 354277)-coated plates. Cells were passaged after 4-5 days.

### Embryoid body and cerebral organoid cultures

The formation of EB: EB is an early embryo analog which can develop into cerebral organoid or other organoids. Initially, hiPSCs were dissociated into single cells with 0.5 mM EDTA (life technologies, 15,575,020) and suspended at density of 4 × 10^7^/L with EB formation media (100ul/per EB sphere). The formation medium (Stem Cell, 08574) was supplemented with Rock Inhibitor (Tocris, Y-27632), fresh media were replaced every other day. After 3-4 days culture, homogeneous cell masses were formed, and a cell mass is named EB.

The development of CO: Specific inducers such as neural signal molecules were added to promote the development of cerebral cortex. Under neural induction, the neural rosettes (NRs) were induced: Initially, several neural rosettes composed of neuroepithelium around central cavity were appeared inside EBs. NR was also an important primordium of CO as neural tube in animals. At day 5, EBs were cultured into low-adhesion 6-well plates, 1.5 ml neuroepithelial induction medium (Stem Cell, 08572) was added to each well, and after 48 hrs, and EBs were replaced with 2 ml neuroepithelial enlargement medium, in a CO2 incubator at 37 °C, with 5% CO2 and balanced humidity for 96 hrs. Then, an EB was transferred to a Matrigel droplet (BD Biosciences, 356,234). The Matrigel droplets were removed to a 60-mm dish with 5 ml of cerebral cortex maturation medium (stem cell, 08573). The cultures were conducted at 37 °C with 5% CO2 and shaking. The culture medium was changed every 2 days, and the results were checked under a reverted microscope. After continuous cultures, the NRs will develop into COs and mature COs could not be found until day 40, with typically laminar structure.

### RSV treatment

EBs with uniform size were randomly divided into three groups including the Control, the D-gal and RSV group. RSV group included RSV-L (2 μm/L), RSV-M (10 μm/L) and RSV-H (50 μm/L). The following is the detail of grouping: (1) control group: the EBs were cultured with regular medium. (2) D-gal group or nervous injury group: the EBs were exposed to 5 mg/ml D-gal (Solarbio, D8310) in medium from day 3, in order to induce nervous injury. (3) RSV groups: after D-gal for 24 hrs, the cultures with the continuation of D-gal were exposed to RSV (Sigma-Aldrich, R5010) in medium with three different doses, such as 2 μm/L, 10 μm/L and 50 μm/L. The cultures with uniform size were harvested at D5, D8, D12 and D40 for follow-up experiments. The number of specimens for each group is greater than or equal to 5 at each age (*N* ≥ 5) (only 5 specimen could be chosen in RSV-H at D40).

### Immunofluorescent staining

EBs and COs were fixed with 4% paraformaldehyde (pH 7.2) for 1 hr. at 4 °C, and they were tissue-protected in 30% of sucrose in PBS overnight at 4 °C, then serial sections (15 μm) were cut with a cryostat, and sections were blocked with 5% normal goat serum for 30 min at room temperature and incubated overnight at 4 °C with different primary antibodies: mouse anti-Sox2 (1:200, Abcam, ab97959); rabbit anti-ki67 (1:100, Abcam, ab15580); rabbit anti-caspase-3 (1:200, Abcam, ab13847); mouse anti-Nestin (1:1200, Stem cell, 60,091); rabbit anti-CXCR4 (1:250, Abcam, ab124824); mouse anti-β-III-tubulin (1:200, Abcam, ab78078); rabbit anti-collagen IV (1:200, Abcam, ab6586). After multiple washes in 0.01 M PBS, appropriate secondary antibodies were added at indicated dilutions and incubated at room temperature for 3 hrs. The secondary antibodies were as follows: Alexa Fluor 488 conjugated with goat anti-mouse IgG (1:600, Invitrogen, A11031); Alexa Fluor 568 conjugated with goat anti-rabbit IgG (1:400, Invitrogen, A11034). After immunoreaction, the sections were cover-slipped with 65% glycerol (in 0.01 M PBS) and 300 nM DAPI nucleic acid stain (Solarbio, C0060) for counterstaining and imaged using an fluorescence microscope (BX53, Olympus) with rhodamine, fluorescein isothiocyanate, or ultraviolet filter sets.

### Measurements and statistical analyses

Quantitating EBs and COs size: The diameters (μm) of EBs and COs were measured under a reverted microscope by ImageView. The percentage of positive cells (%) = the numbers of positive cells/the numbers of total cells × 100%, such as apoptotic cells and immuno-positive cells were calculated, with ImageJ soft (http://rsb.info.nih.gov/ij/). At least five specimens were used for each group at each age, and each specimen was sectioned into 5 photos for measurements.

After the measurements described above, the data were compared using independent-samples t-tests between control and treatment groups. One-way ANOVA with Tukey post-test was used. Graphing was performed with GraphPad Prism software (Prism5.0), all values were represented as means ± S.E.M, and *P* < 0.05 was accepted as statistical significance.

## Data Availability

All data generated or analysed during this study are included in this published article. The datasets used and/or analysed during the current study are available from the corresponding author on reasonable request.

## Abbreviations

hiPSCs: Human induced pluripotent stem cells
hESC: Human embryonic stem cells
RSV: Resveratrol
EBs: Embryoid bodies
COs: Cerebral organoids
3D: 3 dimensional
D-gal: D-galactose
NSCs: Neuroepithelial stem cells
NRs: The neural rosettes
RSV-L: The RSV low-dose group
RSV-M: The RSV medium-dose group
RSV-H: The RSV high-dose group
DAPI: 4,6-diamino-2-phenylindole
CXCR4: C-X-C Motif Chemokine Receptor 4
Cdx2: Caudal Type Homeobox 2
SIRT1: Class III histone deacetylase sirtuin1
